# An orbitally derived single-atom magnetic memory

**DOI:** 10.1038/s41467-018-06337-4

**Published:** 2018-09-25

**Authors:** Brian Kiraly, Alexander N. Rudenko, Werner M. J. van Weerdenburg, Daniel Wegner, Mikhail I. Katsnelson, Alexander A. Khajetoorians

**Affiliations:** 1 Institute for Molecules and Materials, Radboud University, Nijmegen, 6525 The Netherlands; 2 School of Physics and Technology, Wuhan University, 430072 Wuhan, China; 3 Theoretical Physics and Applied Mathematics Department, Ural Federal University, Ekaterinburg, Russian Federation 620002

## Abstract

A magnetic atom epitomizes the scaling limit for magnetic information storage. Individual atomic spins have recently exhibited magnetic remanence, a requirement for magnetic memory. However, such memory has been only realized on thin insulating surfaces, removing potential tunability via electronic gating or exchange-driven magnetic coupling. Here, we show a previously unobserved mechanism for single-atom magnetic storage based on bistability in the orbital population, or so-called valency, of an individual Co atom on semiconducting black phosphorus (BP). Ab initio calculations reveal that distance-dependent screening from the BP surface stabilizes the two distinct valencies, each with a unique orbital population, total magnetic moment, and spatial charge density. Excellent correspondence between the measured and predicted charge densities reveal that such orbital configurations can be accessed and manipulated without a spin-sensitive readout mechanism. This orbital memory derives stability from the energetic barrier to atomic relaxation, demonstrating the potential for high-temperature single-atom information storage.

## Abstract (web summary)

Single-atom storage can dramatically exceed the limit of traditional high-density memory devices but is challenging. Here the authors show the bistability in the orbital configuration of a single Co atom on black phosphorus which can be accessed, manipulated and has potential for high-temperature single-atom information storage.

## Introduction

Single-atom memory represents the ultimate limit in high-density storage^1–3^ and a route toward quantum coherent manipulation^4,5^. Of particular interest are single magnetic atoms on surfaces, which can represent a bit employing the bistability of the magnetic moment, as they offer tunable interatomic coupling and bottom-up design^6^. While atomic spins can have long lifetimes^2,3,7^, the key challenge has been to decrease fluctuations induced by spin-sensitive readout or scattering mechanisms^8,9^ utilizing robust magnetic anisotropy^2^. The strategy toward single-atom magnetic memory has largely been to utilize certain combinations of atoms and surfaces in which the moment-bearing orbitals responsible for magnetism weakly hybridize with the environment^3,7^. Ultimately, this limits the selective coupling between neighboring atoms^10^ and electronic access to the spin^11^. Single dopants in semiconductors^12^ offer an attractive route toward atomic-scale memory^4^ and processing^13,14^, with the advantage of gating while still weakly hybridizing with the localized spin^15^. Scanning tunneling microscopy (STM) of individual non-magnetic dopants^16–18^ and dangling bonds^19^ revealed bistability of ionization states and has been used to study the interplay between local screening and confinement^20,21^. However, the magnetic properties of individual magnetic impurities in semiconductors^15^ have been poorly explored compared to their counterparts on metals and thin-film insulators^22,23^.

Black phosphorus (BP) is a particularly intriguing semiconductor and tunable Dirac material^24,25^, with a strongly anisotropic band structure and thickness-dependent band gap^26^. To date, the magnetic properties of individual dopants in BP have not been experimentally investigated, in contrast to other Dirac materials^27,28^. Here, we demonstrate a single-atom memory derived from bistability of an individual Co atom on semiconducting BP. Utilizing STM and ab initio calculations, we visualize and identify the spatially anisotropic charge densities of each state; the calculations reveal screening-stabilized orbital repopulation as the origin of bistability and further indicate unique magnetic moments for each of the valencies. We experimentally detail the effect of the local tip-induced gate potential on the switching behavior between the two. This opens up the possibility of utilizing the orbital degree of freedom for robust single-atom magnetic information storage without requiring spin-sensitive detection, as well as understanding the effect of local gating on the anisotropic charge distribution of a single atomic bit.

## Results

### Cobalt deposition and manipulation

**Fig. 1:**
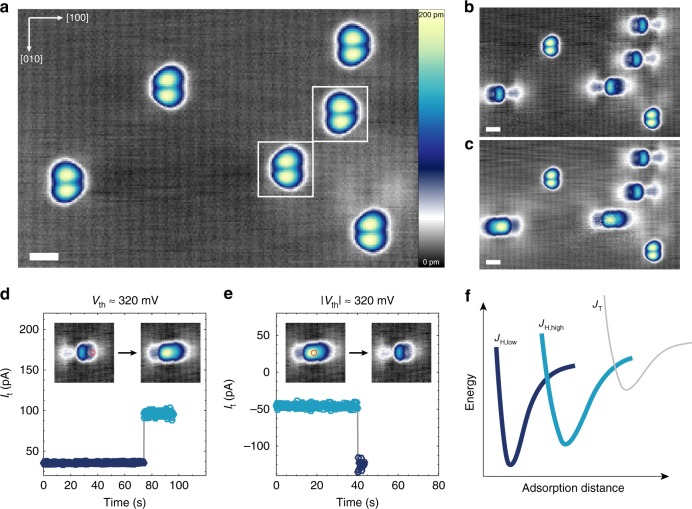
Adsorption and switching of Co on BP. **a** Six Co species on BP as deposited at *T* < 5 K (*V*_s_ = −400 mV, *I*_t_ = 20 pA, scale bar = 1 nm). Boxed atoms show species related through mirror plane along [010]. **b** Four atoms from **a** have been switched into *J*_H,low_ (*V*_s_ = −400 mV, *I*_t_ = 20 pA, scale bar = 1 nm). **c** Two atoms from **b** have been switched into *J*_H,high_ (*V*_s_ = −400 mV, *I*_t_ = 20 pA, scale bar = 1 nm). **d** Switching characteristics from *J*_H,low_ to *J*_H,high_ with *V*_s_ = 420 mV and **e** *J*_H,high_ to *J*_H,low_ with *V*_s_ = −680 mV. Approximate threshold biases for switching (*V*_th_) are noted. Orange circles indicate the tip position during the switching sequence. The inset images showing before and after configurations are 4 nm × 4 nm in size. **f** Schematic representation of adsorption energy curves for Co species on BP

The result of Co deposition on a BP surface cleaved in situ is shown in Fig. 1a, where the surface illustrates the expected buckled rhombohedral structure. Individual clean Co atoms are identified as bi-lobed butterfly-like shapes due to the anisotropic extension of their charge density upon adsorption onto BP (see Supplementary Figure 1 for larger area images before and after deposition and Supplementary Figure 2 for analysis on the presence and influence of hydrogen). As seen in Fig. 1a, two types of bi-lobed Co species are observed (boxed atoms Fig. 1a), related through mirror symmetry along the zig-zag [010] direction, similar to single vacancies in BP^29^. High-resolution analysis of the STM data (Supplementary Figure 3) reveals that the bi-lobed species reside on top sites. These species account for approximately ~98% of the as-deposited atoms, indicating favorability toward top-site adsorption during low-temperature (*T* ≈ 5 K) deposition; here, the areal density (Fig. 1a) is approximately 0.022 ± 0.003 nm^−2^ (see Supplementary Figure 1).

**Fig. 2:**
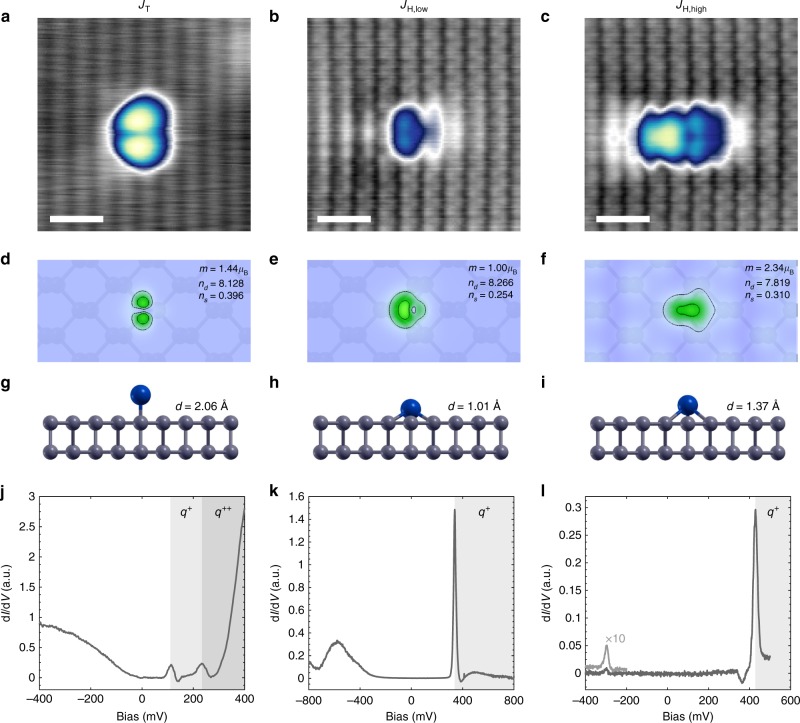
Ground states of Co atoms. High-resolution image of Co in **a** *J*_T_ (*V*_s_ = −400 mV, *I*_t_ = 200 pA, scale bar = 1 nm), **b** *J*_H,low_ (*V*_s_ = −60 mV, *I*_t_ = 200 pA, scale bar = 1 nm), and **c** *J*_H,high_ (*V*_s_ = −60 mV, *I*_t_ = 200 pA, scale bar = 1 nm) configurations with same color scale as Fig. 1. DFT calculations of charge density distributions, including magnetic moment (*m*), *n*_*d*_, and *n*_*s*_, for **d** Co on a top site, **e** Co in a hollow site, and **f** Co in a hollow site with *U* = 4 eV. **g**–**i** Schematics of relaxed atomic adsorption geometries with out-of-plane distance (*d*) noted. **j**–**l** d*I*/d*V* spectra taken on each atom

Upon current injection with the STM tip above a voltage threshold (Fig. 1d), individual Co atoms can be manipulated from the top site to a hollow site (Fig. 1b and Supplementary Figure 4), as confirmed by atomic resolution imaging (Supplementary Figure 3). This shift of binding site involves a clear modification to the spatial charge density distribution. Surprisingly, we find that there are two unique and stable shapes of the Co atom within the same hollow site (Figs. 1c, d and 2), as exhibited by the variation in the charge density. We denote these two states as *J*_H,low_ and *J*_H,high_ (index H denotes the atomic site and high/low refers to the size of the magnetic moment shown in Fig. 2e, f). In addition to their unique spatially distributed charge density, *J*_H,high_ can also be distinguished by its larger apparent height in STM constant-current images (*J*_H,high_ = 176 ± 8 pm, *J*_H,low_ = 132 ± 4 pm at *V*_s_ = −400 mV). Switching between *J*_H,low_ and *J*_H,high_ was achieved via location-dependent current injection (Fig. 1d, e), with *J*_H,high_ to *J*_H,low_ at |*V*_s_|  ≳ 320 mV and *J*_H,low_ to *J*_H,high_ at *V*_s_ ≳ 320 mV. Notably, the switching between different hollow-site states is fully reversible, as shown in Supplementary Figure 4. However, once a Co atom is manipulated into the hollow site, we were not able to relocate it back into a top site (denoted *J*_T_, cf. Fig. 1f). Each of the three atomic configurations remained stable (as probed for measurement times up to 17 h) until intentionally perturbed. Furthermore, unlike charge switching in single dopants on semiconductors, the atomic state remains fixed after removing the applied bias^16^ and the charging lifetime is expected to be very short due to the strong native *p*-doping of the BP crystal^29^.

### Ab initio calculations and state identification

To elucidate the origin of each experimentally observed Co state, we performed density functional theory (DFT) calculations for a Co atom residing on a top (Fig. 2d) and hollow site (Fig. 2e, f) to compare with experimental data (Fig. 2a–c). The calculations were carried out for monolayer BP under the generalized gradient approximation (GGA); to include the effects of local Coulomb interactions in the Co 3*d* orbital, calculations involving a Hubbard-U correction (GGA + U method) were also performed. Varying the Hubbard-U parameter (Supplementary Figure 5) reveals the mutually exclusive stability of two unique states with a critical value at approximately *U* = 3.5 eV, where the state favorability between *J*_H,low_ and *J*_H,high_ is inverted. Plotting the spatial distribution of the total charge density from the DFT calculations (Fig. 2d–f), we were able to directly associate each calculation to a corresponding constant-current STM image. The qualitative agreement is excellent and enables us to confirm the experimental binding-site analysis and to roughly approximate the effective screening parameter (*U* = 0–3 eV for *J*_T_ and *J*_H,low_, *U* = 4–6 eV for *J*_H,high_—see Supplementary Figure 5) for the Coulomb repulsion of the Co 3*d* orbital. When including relaxation into the hollow-site calculations, we find that the atomic positions in the surface plane are identical (although out-of-plane distances are different—see Fig. 2h, i, and the schematic potential diagrams in Fig. 1f); namely, the experimental switching from *J*_H,low_ to *J*_H,high_ can neither be attributed to a change in binding site nor to different charge configurations.

The use of the Hubbard-U correction allows us to assess distance-dependent screening from the surface within the 3*d* shell of the Co atom. As substrate separation (*d*) is reduced, the more extended 4*s* orbital becomes energetically less favorable due to Pauli repulsion with the BP ligand field, while the increased screening of the 3*d* orbital increases its energetic favorability by decreasing Coulomb repulsion in the system. The resulting occupation of the Co 4*s* (*n*_*s*_) and *3d* (*n*_*d*_) orbitals is given in Fig. 2 for each of the states (resolved into the 3*d* subshells in Supplementary Table 1). We find from these calculations that the relaxation (Δ*d*) from *J*_H,low_ to *J*_H,high_ (Fig. 2h, i) is accompanied by a redistribution of the 4*s*-orbital and 3*d*-orbital populations (for further detail, see Supplementary Figures 6, 7, and 8). As expected, when modifying metal 3*d*-orbital occupancy, the total magnetic moment also changes between 1.00*μ*_B_ for *J*_H,low_ and 2.34*μ*_B_ for *J*_H,high_. Furthermore, calculations of the magnetic anisotropy indicate that the easy axis also changes from in-plane (*J*_H,low_) to out-of-plane (*J*_H,high_) (Supplementary Table 2). This suggests that the magnetic anisotropy of Co can be controlled electrically in this system. We note that similar orbital behavior has been predicted for transition-metal atoms on graphene^30–32^, where multiple states (different *d*) were analogously predicted due to the reorganization of the orbital occupancies. Quantum chemistry calculations for Co on graphene further indicated that the energy barrier between states could reach nearly 300 mV^30^, which might explain the remarkable stability of the states observed here. This also indicates that using the orbital degree of freedom may be much more robust compared to using solely the bistability of the spin ground states.

### Tip-induced local gating

In order to elucidate the valency switching mechanism, we studied the influence of tip-induced band bending (TIBB) on the Co states. Due to insufficient screening from charge carriers, the applied potential between tip and sample locally influences the energy of semiconductor bands; if an impurity level, shifted with the material bands, passes through the Fermi level (*E*_F_), it will undergo an observable charging/discharging event in STM and scanning tunneling spectroscopy (STS)^33^. Such charging events resulting from TIBB can be distinguished by peaks in d*I*/d*V* whose location and intensity are strongly sensitive to the stabilization parameters (Supplementary Figure 9) and tip location (Supplementary Figure 10)^33,34^. While all states demonstrate ionization events, we limit our focus in this work to the *J*_H,low_ and *J*_H,high_ states. A representative d*I*/d*V* spectrum for *J*_H,low_ (Fig. 2k) clearly shows a strong peak at approximately 280 mV, while the primary peak for *J*_H,high_ (Fig. 2l) is seen at 420 mV. In conjunction with the spectroscopic mapping (see below), the shaded regions (labeled *q*^*+*^) are identified as bias ranges where the Co species have been ionized. At biases greater than these thresholds, the atoms are non-locally ionized via the tip-induced potential along the BP surface.

**Fig. 3:**
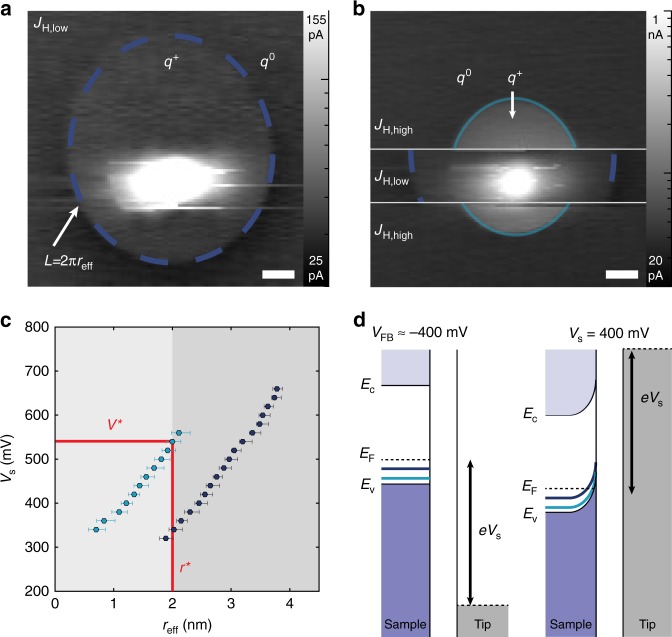
Ionization of Co. **a** Constant-height current map of *J*_H,low_ (setpoint conditions: *V*_s_ = 500 mV, *I*_t_ = 40 pA), showing isotropic charging state (highlighted with dashed blue ring) as a disk in the current map (logarithmic color scale, scale bar = 1 nm). Charged (*q*^+^) and uncharged (*q*^0^) regions are denoted. **b** Constant height map of same Co atom initialized into *J*_H,high_ state (setpoint conditions: *V*_s_ = 500 mV, *I*_t_ = 40 pA, scale bar = 1 nm, logarithmic color scale). The atom switches into *J*_H,low_ for a small section in the middle of the image. **c** Charging ring effective radius (*r*_eff_ = *L*/2*π*) as a function of bias for *J*_H,low_ (dark blue) and *J*_H,high_ (light blue). Red lines show threshold bias (V*) determined from switching data and corresponding critical radius (r*) for *J*_H,low_ and *J*_H,high_. Error bars are derived from experimental uncertainty in the measured disk radius, further explained in Supplementary Figure 11. **d** Schematics of proposed flat-band condition (*V*_FB_ ≈ −0.4 eV) and band bending at selected bias near **a**, **b**

To gain a more complete picture of this local surface potential, we used constant height imaging to map out the spatial dependence of the ionization as a function of bias voltage (Fig. 3a, b)^34^. The size of the isotropic disk (stepwise increase in current around the Co, or the so-called charging ring when imaged in d*I*/d*V* maps—Supplementary Figures 10 and 11) scales similarly for both states with bias according to hyperbolic contours of constant TIBB (Fig. 3c). This indicates equivalent screening from the BP for each Co configuration. Furthermore, the trend of the effective ring radii (*r*_eff_ = *L*/2*π*, where *L* is the ring circumference) with applied bias (see also Supplementary Figure 10) indicates a flat-band condition of *V*_FB_ < −300 mV (Fig. 3d). Such a condition is achievable with a tip work function of 4.0–4.1 eV. Identifying the flat-band condition and the ring-radius dependence on bias indicates that the ionization events are caused by the upward bending of states below *E*_F_ (see Fig. 3d). Theoretical calculations for both configurations reveal non-zero density of states below *E*_F_; however, *J*_H,low_ clearly has a strong 3*d*-orbital peak in the DOS between *E*_F_ and the valence band edge (*E*_v_). Consistently smaller radii for *J*_H,high_ compared to *J*_H,low_ indicate that larger TIBB is needed to ionize *J*_H,high_ (near 400 mV); thus, this state must lie farther from *E*_F_ than the ionized state of *J*_H,low_ (Supplementary Figure 10h).

### Switching dynamics and mechanism

**Fig. 4:**
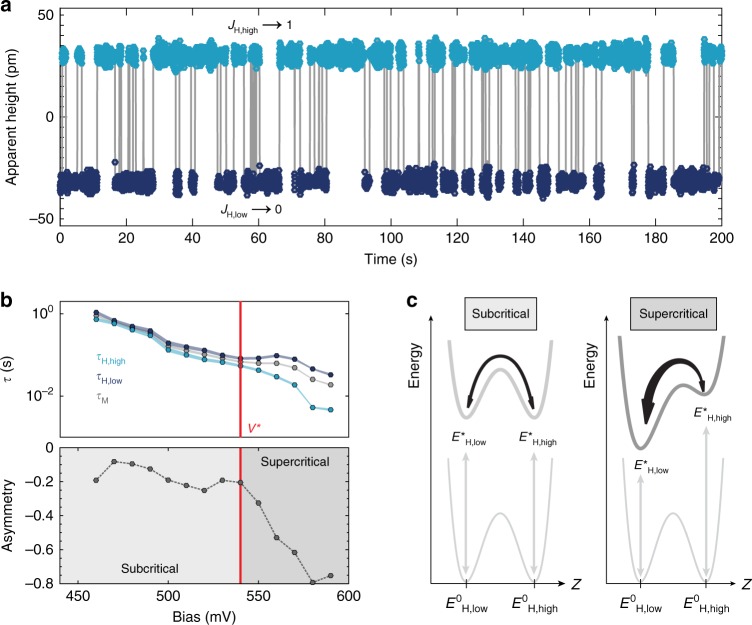
Orbital memory. **a** Two-state conductance signal with *J*_H,high_ (light blue) showing high conductance and *J*_H,low_ (dark blue) showing low conductance. **b** State lifetime and asymmetry as a function of tip bias with tip height held constant. Linewidth of lifetime curves indicates measurement error. V* highlights critical threshold where TIBB causes lifetime depression for *J*_H,high_. **c** Energy schematics illustrating excitation/ionization (*E*^0^ to E*) and switching mechanism for orbital memory. (Left) Sub-threshold behavior with nearly equivalent lifetimes (symmetric upper well structure) and (right) supercritical behavior with strongly diminished *J*_H,high_ lifetime (imbalanced upper well structure)

**Fig. 5:**
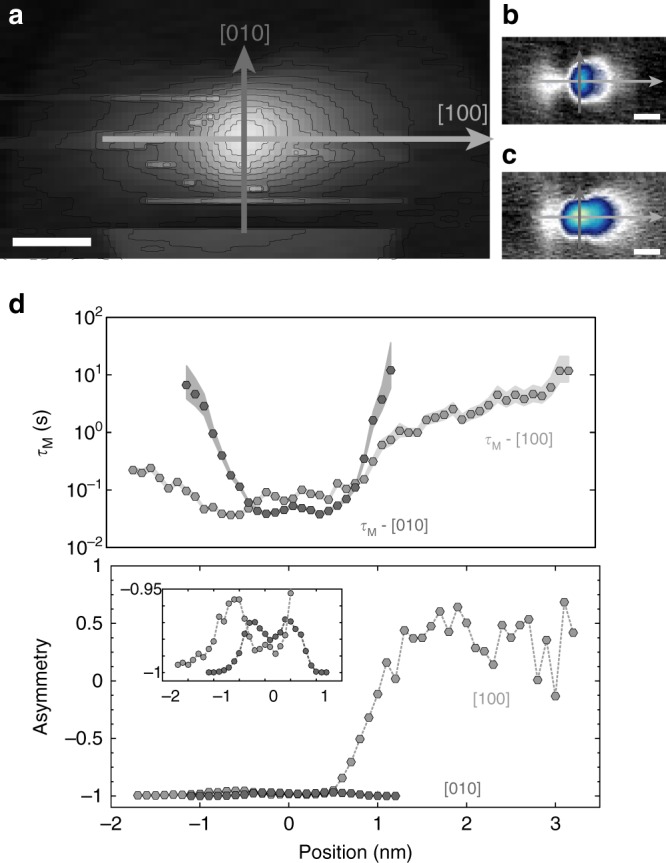
Anisotropic gating. **a** Constant-height current map showing Co charge density at *V*_s_ = 500 mV (setpoint conditions: *V*_s_ = 500 mV, *I*_t_ = 40 pA, scale bar = 1 nm). Dark gray and light gray arrows show measurement positions for **d** along [010] and [100], respectively. **b** Measurement locations shown on constant-current image of *J*_H,low_ (*V*_s_ = −400 mV, *I*_t_ = 20 pA, scale bar = 1 nm). **c** Measurement locations with reference to *J*_H,high_ (*V*_s_ = −400 mV, *I*_t_ = 20 pA, scale bar = 1 nm). **d** Spatially resolved constant height measurements (setpoint conditions: *V*_s_ = 500 mV, *I*_t_ = 40 pA). Mean lifetime (top) and state asymmetry (bottom) as a function of position along (light gray) [100] and (dark gray) [010] directions. (Inset, bottom panel) Rescaled plot of asymmetry data from positions −2 to 1 nm. The constant height image in **a** was taken with precisely the same tip height and bias to show the length scale of the ionized atomic wavefunction (atom is primarily in *J*_H,low_). Position 0 nm corresponds to the center of the Co atom. Data were collected with *V*_s_ = 500 mV

Upon gating the Co into the charged regimes (*q*^*+*^ regions in Fig. 2k, l) with the STM tip, a discrete, bistable conductance signal, or the so-called telegraph noise, is measured on the Co atoms (Fig. 4a). The bistable states are correlated to the *J*_H,low_ (dark blue) and *J*_H,high_ (light blue) configurations of Co via independent constant-current measurements at lower negative biases (−400 mV < *V*_s_ < −200 mV), which do not perturb the respective states. The ability to read and write both Co orbital configurations confirms its utility as a binary memory; thus, we denote *J*_H,low_ as state 0 and *J*_H,high_ as state 1. To further probe the stability and favorability of these states, we studied the bias and spatial dependencies of the telegraph noise. We define *τ* as the residence time for the given state, as derived in ref ^35^. Figure 4b illustrates the effect of bias at constant height. Maintaining a constant tip-sample separation, while smoothly varying the bias, results in a proportional change in the TIBB at the surface. In this manner, the influence of TIBB on the state stability can be directly measured without artifacts introduced by changing the tip-sample distance. As seen in the top panel of Fig. 4b, at lower biases *τ*_H,low_ and *τ*_H,high_ are nearly equivalent and strongly decay with increasing TIBB, resulting from the increased energy and flux of the tunneling electrons. However, above a threshold voltage *V*_s_ *=* V* ≈ 540 mV, the state-dependent lifetimes diverge from each other, leading to a large state favorability or what we define as asymmetry $\left( {A = \left( {\frac{{\tau _{{\mathrm{H}},{\mathrm{high}}} - \tau _{{\mathrm{H}},{\mathrm{low}}}}}{{\tau _{{\mathrm{H}},{\mathrm{high}}} + \tau _{{\mathrm{H}},{\mathrm{low}}}}}} \right)} \right)$ (lower panel Fig. 4b), whereby *τ*_H,high_ is strongly diminished. This divergence indicates that given sufficient gating (above the critical bias threshold V*), there is a strong energetic favorability in the decay mechanism from *J*_H,high_ to *J*_H,low_ (Fig. 4c). Reexamining the charging ring data from Fig. 3c, we see that this critical bias corresponds to a *J*_H,high_ critical charging ring radius (r*) of approximately 2 nm. We also note here that the onset of telegraph switching occurs after the ring radius for *J*_H,low_ exceeds r*. These observations suggest that a minimum gate potential (measured as a ring radius r*) is required to achieve efficient switching for both states. This threshold is likely related to the extension of the ionized Co charge density, which can span 2–4 nm (see Fig. 5a), as the screening for both states is nearly identical. Based on these observations, we sketch the qualitative energy diagram for the subcritical (*r*_eff_ *<* r*, Fig. 4c left panel) and supercritical (*r*_eff_ *>* r*, right panel) regimes; significantly, the only observed barrier modification is the one between ionized species (E*).

## Discussion

Finally, to understand the connection between the TIBB and the atomic charge density, including the subsequent impact on the switching behavior, we studied the sensitivity of the telegraph noise to the precise tip-gate position (Fig. 5). The upper panel of Fig. 5d shows the mean lifetime (*τ*_M_ = (*τ*_H,high_ + *τ*_H,low_)/2) as a function of position across the Co atom (at constant height) along two orthogonal directions (shown with darker gray ([010]) and lighter gray ([100]) arrows in Fig. 5a–c). The curves reveal contrasting results: along [100], the switching persists anisotropically to a distance of ~3.5 nm from the atom, while the switching rate decays symmetrically and significantly faster along [010] (dark gray). The differences are further reflected in the asymmetry of the states along the two distinct directions, where the zig-zag direction shows almost complete suppression of *τ*_H,high_ (inset, Fig. 5d). The spatial dependencies of Fig. 5d closely match the anisotropy of the *J*_H,low_ ionized charge density (Fig. 5a), pointing to a switching mechanism based on the overlap between TIBB and the spatial extent of the charge density. We note that this switching anisotropy can be utilized to reach almost 100% directed switching probability, which is an important requirement for controlled writing of the single-atom memory.

In conclusion, we utilize a combination of atomic-scale STM/STS and DFT calculations to deduce both the valency and magnetic moment of an individual bistable Co atom at the surface of BP. We show experimentally that the atom can be switched electrically, and is an extremely robust means to store information. DFT shows excellent agreement between charge density plots and the experimentally measured STM images, further revealing that the discrete and stable states result from a shift in the relative orbital population between 4*s* and 3*d* states of the Co atom, which concomitantly changes the spin and magnetic moment. The multiple valencies are stabilized by a change of the effective Coulomb screening between the Co atom and the BP surface. Small differences in the charge and electric dipole nature between the two states could be further elucidated with atomic-scale Kelvin probe experiments to qualitatively probe the valency of each state^36^. Utilizing bistability in the orbital degree of freedom presents several advantages compared to single magnetic atom spin-based computing^3,35^. First, these orbital states can be both read and written electrically without spin sensitivity. Future spin-sensitive detection schemes would provide valuable insight into the spin characteristics of each valency, enabling a means to further explore the behavior of spins in semiconductors and electrically switchable magnetic anisotropy. Second, bistability of the spin moment in each valency would provide four distinct states within each Co atom, thus paving the way for applications toward multi-bit registers. Third, this work also sheds light on the effect of orbital switching by local gating of individual dopants with an anisotropic charge density. Unlike previous studies of single hydrogenic impurities in semiconductors, this work reveals the significance of both TIBB and wavefunction anisotropy in the stability of a two-state system. Our calculations also show that the orientation of the magnetic anisotropy and its amplitude are significantly modified for each of the bistable valencies, indicating a method of electrically controlling the magnetic anisotropy. This motivates future experiments based on spin-resolved STM and inelastic tunneling spectroscopy, which may reveal the nature of the magnetic anisotropy^15^ as well as the spin lifetimes of each Co valency^7^. Finally, the energy separation between orbitals can be significantly larger than magnetic anisotropy or Zeeman energies, potentially making it viable for room temperature application. As single Co atoms were also observed after annealing the sample to room temperature (Supplementary Figure 12), this may open the possibility for realistic higher temperature applications; however, subsequent experiments revealing the energy barriers between the two states will prove pivotal to ascertaining the potential value of such a system for room temperature information storage.

## Methods

### Scanning tunneling microscopy/scanning tunneling spectroscopy

STM/STS measurements were performed in ultrahigh vacuum ( < 1 × 10^−10^ mbar) on an Omicron low-temperature STM with a base temperature of 4.4 K, with the bias applied to the sample. Electrochemically etched W tips were used for measurements; the tips were treated in situ by electron bombardment and field emission, as well as dipped and characterized on a clean Au surface. STS was collected using a lock-in technique to directly measure d*I*/d*V*, a modulation frequency of *f*_mod_ = 4.2 kHz and amplitude of *V*_mod_ = 2–6 mV were applied to the bias signal. BP crystals were provided by HQ graphene and subsequently stored in vacuum ( < 1 × 10^−8^ mbar) at a temperature <25 °C. The crystals were cleaved under ultrahigh vacuum conditions at pressures below 2 × 10^−10^ mbar, and immediately transferred to the microscope for in situ characterization. Cobalt was evaporated directly into the STM chamber with *T*_STM_ < 5 K for the entire duration of the dosing procedure.

### Theoretical calculations

DFT calculations were carried out using the projected augmented-wave method^37^ as implemented in the Vienna ab initio simulation package^38,39^. Exchange and correlation effects were taken into account within the spin-polarized GGA in the parametrization of Perdew–Burke–Ernzerhof^40^. Additional Hubbard-U correction was applied to the 3*d* shell of Co within the GGA + U method^41^ in order to capture the effect of the distance-dependent Coulomb screening. An energy cutoff of 300 eV for the plane-wave basis and the convergence threshold of 10^–6^ eV were used in the self-consistent solution of the Kohn–Sham equations, which checked to be sufficient to obtain numerical accuracy. Pseudopotentials were taken to include 3*s* and 3*p* valence electrons for P atom, as well as 3*s*, 3*p*, and 3*d* valence electrons for Co atom. BP surface was modeled in the slab geometry by a single BP layer with dimensions (3*a* *×* 4*b*) ≈ (13.1 × 13.3) Å with atomic positions fixed to the experimental parameters of bulk BP^42^. Vertical separation between the layers was set to 20 Å. The Brillouin zone was sampled in two-dimensions by a uniform distribution of **k**-points on a (8 × 8) mesh. The position of Co atom was relaxed considering different surface sites (top and hollow) as starting points. We checked that the inclusion of two additional BP layers in the slab does not significantly affect the results presented in the main part. The primary difference between the single-layer and three-layer slabs is the reduction of a gap between the valence and conduction BP states. The behavior of Co atom remains virtually unchanged including the adsorption distances, charge density distribution, and magnetic moments. The charge density distributions shown in Fig. 2d–f were obtained by averaging the total charge density over the energy interval of ~0.3 eV in the valence band edge. The projection of the electronic bands on specific atomic states was done using the formalism of maximally localized Wannier functions^43^ implemented in the wannier90 package^44^.

## Electronic supplementary material

Supplementary Information

## Data Availability

The data from this work can be obtained from the corresponding author upon reasonable request.
