# Supplementary material for: An orbitally derived single-atom magnetic memory
Source: Nat Commun. 2018 Sep 25;9:3904. doi: 10.1038/s41467-018-06337-4 (PMC6156418; doi:10.1038/s41467-018-06337-4)
Supplement: Supplementary file 1 — Supplementary Information [file 41467_2018_6337_MOESM1_ESM.pdf]

Supplementary Information

# An orbitally derived single-atom magnetic memory

Brian Kiraly[1], Alexander N. Rudenko[2,1,3], Werner M.J. van Weerdenburg[1], Daniel Wegner[1], Mikhail I. Katsnelson[1], Alexander A. Khajetoorians[1]

1. *Institute for Molecules and Materials, Radboud University, Nijmegen, The Netherlands*
2. *School of Physics and Technology, Wuhan University, Wuhan 430072, China*
3. *Theoretical Physics and Applied Mathematics Department, Ural Federal University, 620002 Ekaterinburg, Russia*

Table of Contents

Supplementary Note 1: Cobalt deposition, identification, and manipulation on black phosphorus .............. 2

*Supplementary Figure 1. Deposition of Co on BP.* .......... *2*

*Supplementary Figure 2. Characterization of hydrogen contamination.* .......... *4*

*Supplementary Figure 3. Atomic resolution analysis of binding site.* .......... *6*

*Supplementary Figure 4. Full switching sequence.* .......... *7*

Supplementary Note 2: Ab Initio calculations for cobalt/black phosphorus density of states and band structure .......... 8

*Supplementary Figure 5. Calculations with varying Hubbard-U correction.* .......... *9*

*Supplementary Figure 6. Electronic density of state calculations.* .......... *10*

*Supplementary Figure 7. Band structure of Co on BP.* .......... *11*

*Supplementary Table 1. Projections onto the cubic harmonics.* .......... *12*

*Supplementary Figure 8. Out-of-plane charge distribution.* .......... *13*

*Supplementary Table 2. Anisotropy Calculations.* .......... *14*

Supplementary Note 3: Comparison of d*I*/d*V* spectroscopy with density of states calculations .......... 14

*Supplementary Figure 9. Setpoint-dependent point spectroscopy.* .......... *15*

Supplementary Note 4: Tip-induced ionization .......... 15

*Supplementary Figure 10. Charging ring maps.* .......... *17*

Supplementary Note 5: Room temperature annealing .......... 19

*Supplementary Figure 12. Room temperature annealing.* .......... *20*

Supplementary References .......... 21

## Supplementary Note 1: Cobalt deposition, identification, and manipulation on black phosphorus

Supplementary Figure 1 shows constant-current scanning tunneling microscopy (STM) images of the black phosphorus (BP) surface before (Supplementary Figure1a) and after (Supplementary Figure1b) deposition of Co atoms. To avoid Co deposition on the STM tip, the tip was fully retracted during the dose; thus the images do not reflect the exact same sample area. A characteristic vacancy is seen in the constant-current image in Supplementary Figure1a, with a similar vacancy in Supplementary Figure 1b for comparison; both images are shown with the same color scale for apparent height. As described in the main text and seen in Supplementary Figure 1b, approximately 98% of the cobalt atoms initially appear as bi-lobed species (labeled $J_T$) with an apparent height of approximately 180 pm. The coverage shown in Supplementary Figure 1b is 0.026 nm$^{-2}$. For detailed studies of the switching dynamics, the coverage was reduced by approximately an order of magnitude to ensure minimal influence from non-local interactions.

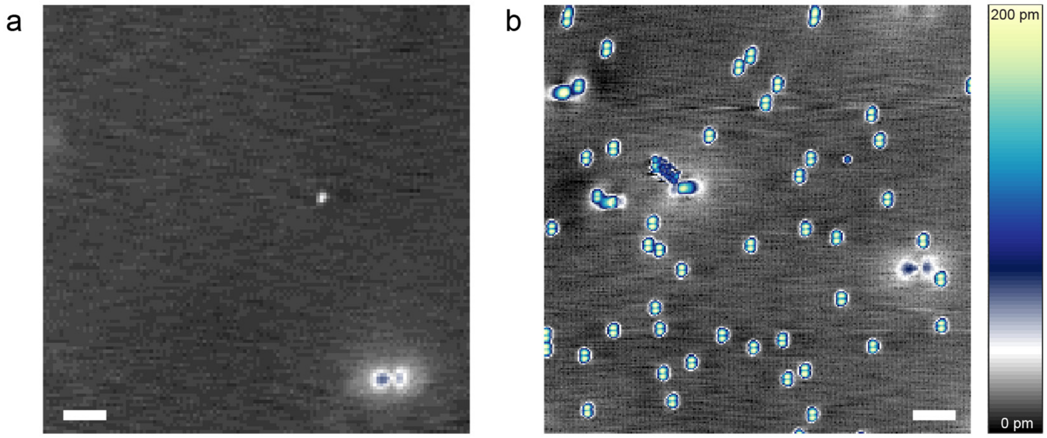


**Supplementary Figure 1. Deposition of Co on BP.** (a) Black phosphorus crystal after cleaving and inserting into STM ($V_s$ = -400 mV, $I_t$ = 20 pA, scale bar = 4 nm). (b) Surface immediately after deposition of Co atoms at $T$ = 5 K ($V_s$ = -400 mV, $I_t$ = 20 pA, scale bar = 4 nm).

It is well known that hydrogen can have a significant impact on the properties of atoms[1-3]. For Co on BP, we show that the addition of a hydrogen atom or atoms to $J_T$ (denoted $CoH_x$, where x

is an unknown integer) causes (left panel, Supplementary Figure 2a): (1) a reduction of the apparent height ($CoH_x \approx 140$ pm) and (2) a modification to the spatial charge density. The species shown in the leftmost panel of Supplementary Figure 2a is hypothesized to be $CoH_x$ because it appears slowly over time (Supplementary Figure 2b-e) and can be irreversibly modified with tip-induced manipulation into $J_T$. This is currently the only species displaying such behavior, consistent with previous reports describing hydrogen desorption from adatoms[2,4]. As shown in Supplementary Figure 2a, after removing the hydrogen, the atomic switching behavior ($J_T$ to $J_{H,low}$ and $J_{H,low}$ to $J_{H,high}$) is as expected, signaling the return of the Co atom to its pristine state. The density of such $CoH_x$ species on a single sample (held at $T$ = 4 K) over the period of 40+ days is shown in Supplementary Figure 2e. In order to minimize the influence of hydrogen, two procedures were used: (1) the cryostat was regularly warmed to $T$ > 40 K and the system pumped with a turbomolecular pump to remove adsorbed hydrogen from the cryostat, and (2) samples were generally studied for less than 10 days to minimize hydrogen contamination. Control spectra were routinely taken on the BP to ensure no spurious features were observed. Finally, the reported spectroscopic features in Fig. 2 and Supplementary Figure 9 were observed with multiple unique tips and did not vary with time.

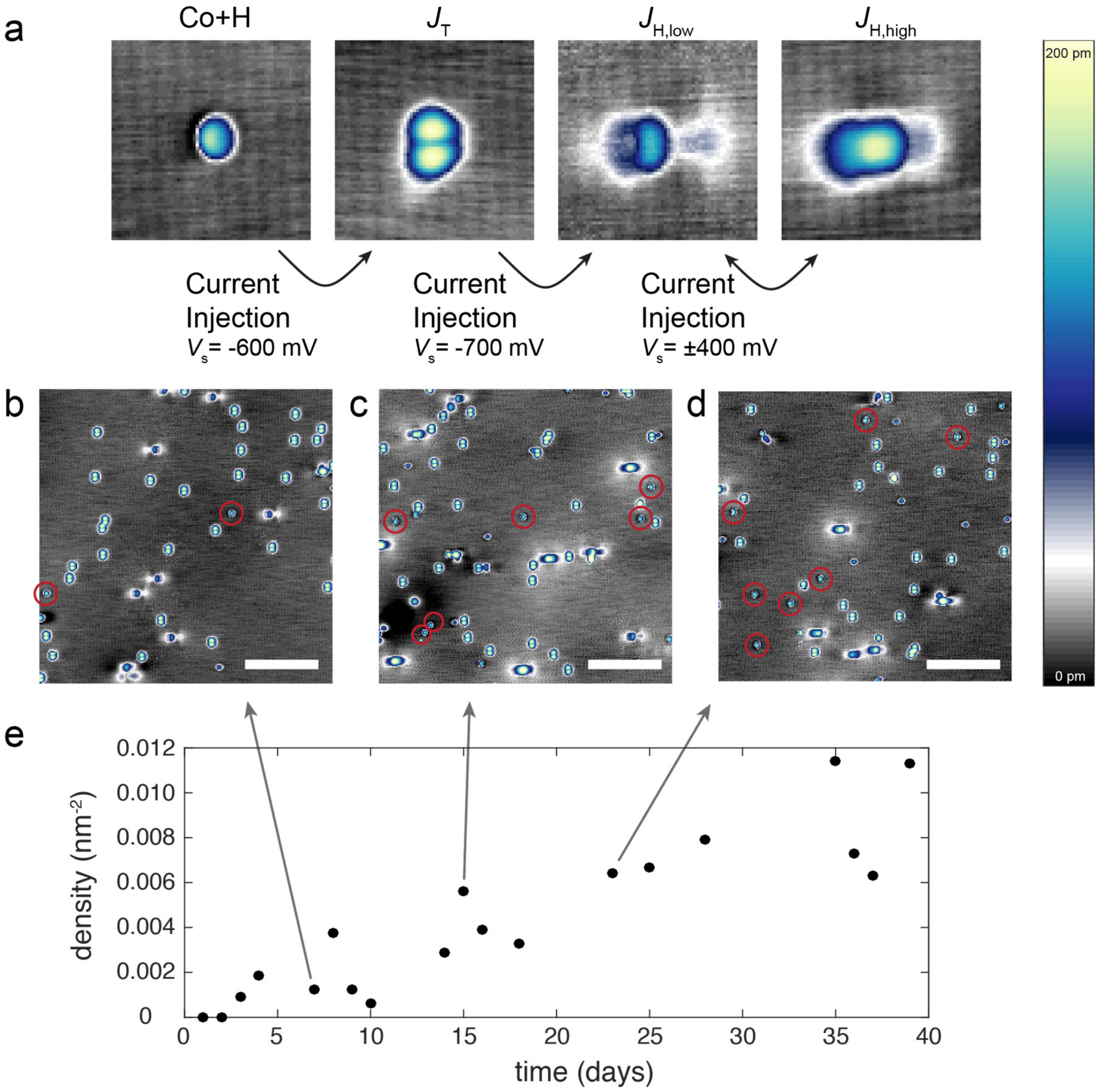


**Supplementary Figure 2. Characterization of hydrogen contamination.** (a) Hydrogenated Co species (red circles in (b), (c), and (d)) tentatively identified by the ability to manipulate them back into the $J_T$ state (second panel). Expected switching behavior ($J_{H,low}$ third panel and $J_{H,high}$ fourth panel) indicates a return of the Co to a pristine state. All images are 4 nm x 4 nm ($V_s$ = -400 mV, $I_t$ = 20 pA). (b), (c) and (d) show STM images of the sample at 7, 15, and 23 days after deposition, respectively ($V_s$ = -400 mV, $I_t$ = 20 pA, scale bar = 4 nm). (e) Density of $CoH_x$ species other than $J_T$, $J_{H,low}$, and $J_{H,high}$ on a single sample held at 4 K for over 40 days.

Binding site analysis was done via atomic resolution imaging at sample biases near the valence band edge (generally -100 mV < $V_s$ < -30 mV) to clearly resolve all P atoms in the upper zig-zag row of the BP surface layer. Unfortunately, at the optimal conditions for BP atomic resolution ($V_s$ = -30 mV, $I_t$ = 20 pA), the $J_T$ state was highly unstable, either shifting binding sites or switching

into $J_{H,low}$. Thus, a sequence of images was used to determine the $J_T$ binding site (Supplementary Figure 3a-c). First, an image was taken at $V_s$ = -400 mV to determine the atomic position (color image Supplementary Figure 3a). The second, partial image was then taken at $V_s$ = -30 mV to determine the BP lattice sites (overlaid image Supplementary Figure 3a). Finally, a third image was taken at $V_s$ = -400 mV to recheck the atomic position. Using the red contours of constant charge density (Supplementary Figure 3b,c), the geometric center of the atom was determined to reside on a phosphorus top site, as indicated in Supplementary Figure 3c. Atomic resolution of the $J_{H,low}$ and $J_{H,high}$ states was acquired simultaneously with the BP lattice (Supplementary Figure 3d,g) despite occasional switching events (Supplementary Figure 3d). Analysis of the constant charge-density contours (Supplementary Figure 3e,h) shows that both species' geometric center resides within a hollow site. Furthermore, the binding site data (Supplementary Figure 3d-j) confirms that both the $J_{H,low}$ and $J_{H,high}$ states for a single atom reside in the same hollow site. Finally, the contours of constant charge density (Supplementary Figure 3c,f,j) for all three states agree well with the DFT charge density predictions given in Fig. 2j-l, further confirming the binding site assignment.

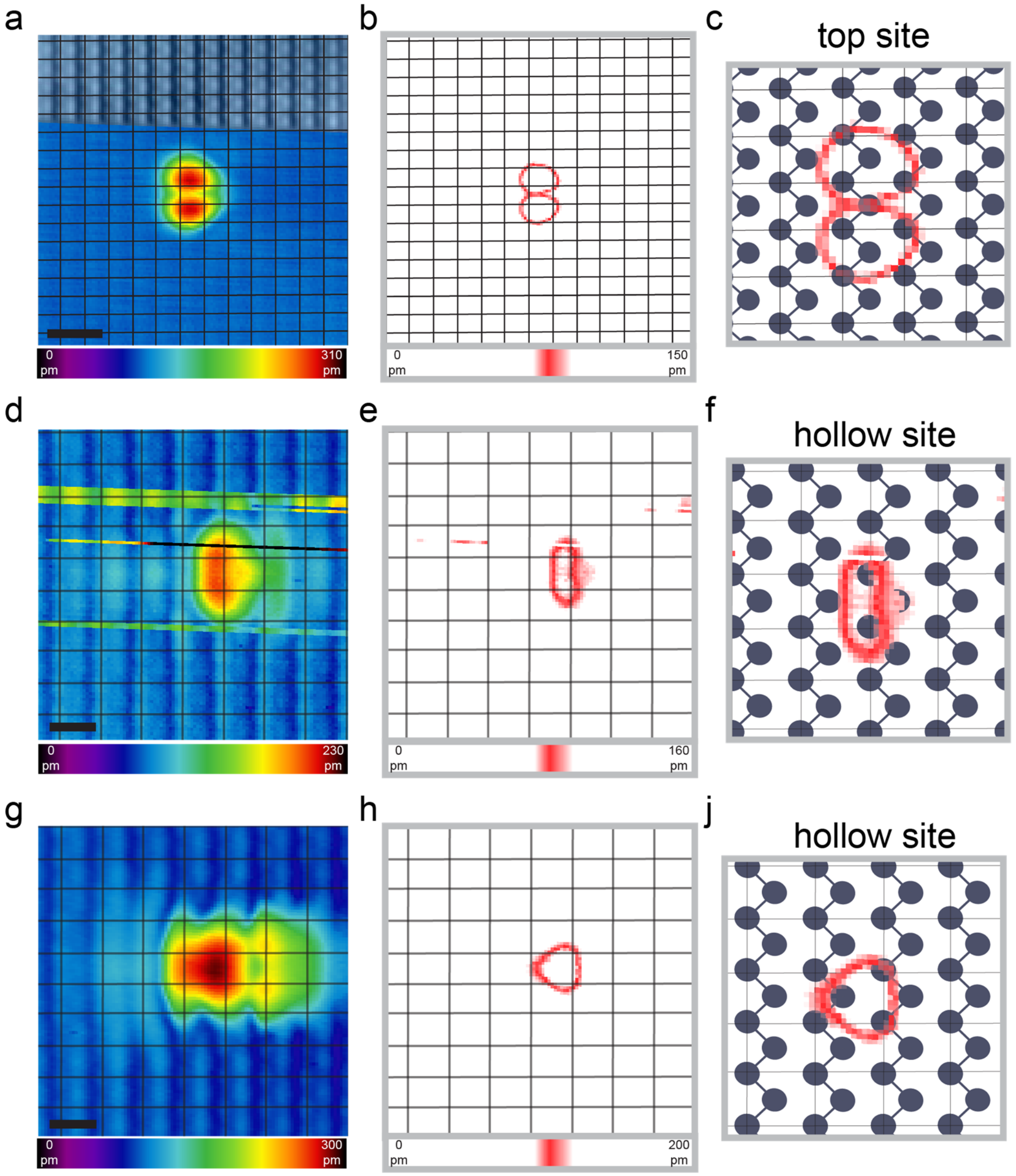


**Supplementary Figure 3. Atomic resolution analysis of binding site.** (a) Top-site atom with sublattice-resolved image overlaid on an adatom image (adatom image: $V_s$ = -400 mV, $I_t$ = 20 pA, scale bar = 1 nm, sublattice image: $V_s$ = -30 mV, $I_t$ = 20 pA). The black phosphorus conventional unit cell (black – reflecting the periodicity of one of the two sublattices) as determined by the sublattice-resolved image is overlaid on the STM data. (b) STM data from (a) is replotted to select a single range of apparent height (charge density) to clearly denote the location of the Co on the BP lattice. (c) Binding site analysis using the red contour of constant charge density to determine the geometric center of the atom. The actual BP lattice has been included for clarity based on the unit cell from (a,b). (d) Atomic resolution image of $J_{H,low}$ state with sublattice resolution of the BP ($V_s$ = -30 mV, $I_t$ = 20 pA, scale bar = 0.5 nm). Again, the BP conventional unit cell is overlaid in black. (e) Contour of constant charge density plotted with the BP lattice. (f) Hollow binding site designation based on the constant charge density contour. (g) Atomic resolution image of $J_{H,high}$ state with sublattice resolution ($V_s$ = -30 mV, $I_t$ = 20 pA, scale bar = 0.5 nm). Black phosphorus lattice overlaid in black. (h) Contour of constant charge density plotted with the BP lattice. (j) Hollow binding site designation based on the constant charge density contour. All images have been corrected for a 2° offset between the x and y directions.

A step-wise switching sequence, showing both the localization and reversibility of the switching, is given in Supplementary Figure 4. Supplementary Figure 4b-e show the switching of atoms 1-4 (Supplementary Figure 4a), respectively, from the $J_T$ state into the $J_{H,low}$ state. The isolated manipulation of atoms 2-4 (Supplementary Figure 4c-e) despite their ~ 2 nm separation, demonstrates the confinement of the tip-induced perturbation responsible for the switching. Supplementary Figure 4f,g further shows the switching of atoms 1and 2 from the $J_{H,low}$ state into the $J_{H,high}$ state. To demonstrate the reversibility of the $J_{H,low}$ to $J_{H,high}$ transition, Supplementary Figure 4h,j show the reverse switch from the $J_{H,high}$ state back into the $J_{H,low}$ state. As seen in the telegraph data from Fig. 4, reversible switching between $J_{H,high}$ and $J_{H,low}$ was observed 1000's of times without modification to the constituent states.

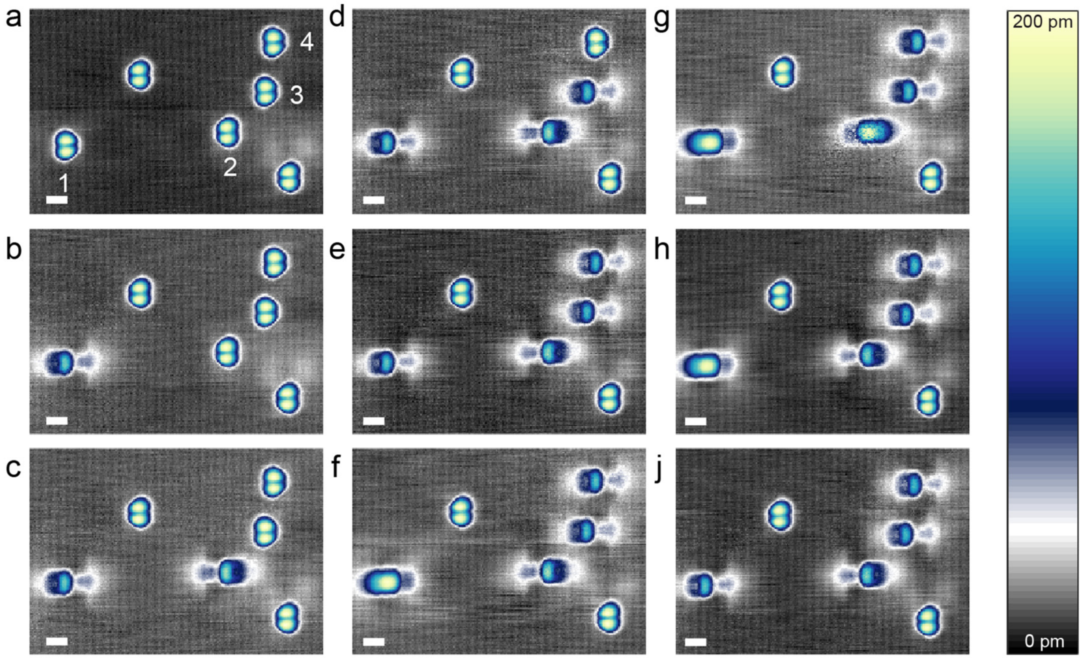


**Supplementary Figure 4. Full switching sequence.** (a) Initial state for all six species. (b-e) Switching atoms 1-4 from $J_T$ site to $J_{H,low}$. (f,g) Switching atoms 1-2 from $J_{H,low}$ into $J_{H,high}$. (h,j) Switching atoms 1-2 from $J_{H,high}$ into $J_{H,low}$. All images: $V_s$ = -400 mV, $I_t$ = 20 pA, scale bar = 1 nm.

## Supplementary Note 2: Ab Initio calculations for cobalt/black phosphorus density of states and band structure

Supplementary Figure 5 shows total energies of the $J_{H,low}$ and $J_{H,high}$ states calculated as a function of the Hubbard-U parameter. One can see that for $U < 3.5$ eV the $J_{H,low}$ state is energetically favorable, whereas at $U > 3.5$ eV the $J_{H,high}$ state becomes more stable. The corresponding charge distribution averaged over the valence band edge (as in Fig. 2d-f) are shown at the bottom of Supplementary Figure 5. In these panels, it is clear that the shape of the charge distribution is different for the two orbital states, irrespective of the exact value of the Hubbard-U parameter for each state. In this theoretical analysis, the Hubbard-U is a phenomenological parameter related to the Coulomb repulsion. To adequately describe two distinct orbital configuration of Co on BP it is therefore sufficient to consider $U$=0-3 eV for $J_{H,low}$ and $U$=4-6 eV for $J_{H,high}$. For comparison with experimental STM images, we use $U$=0 and $U$=4 eV for the $J_{H.low}$ and $J_{H,high}$ states, respectively.

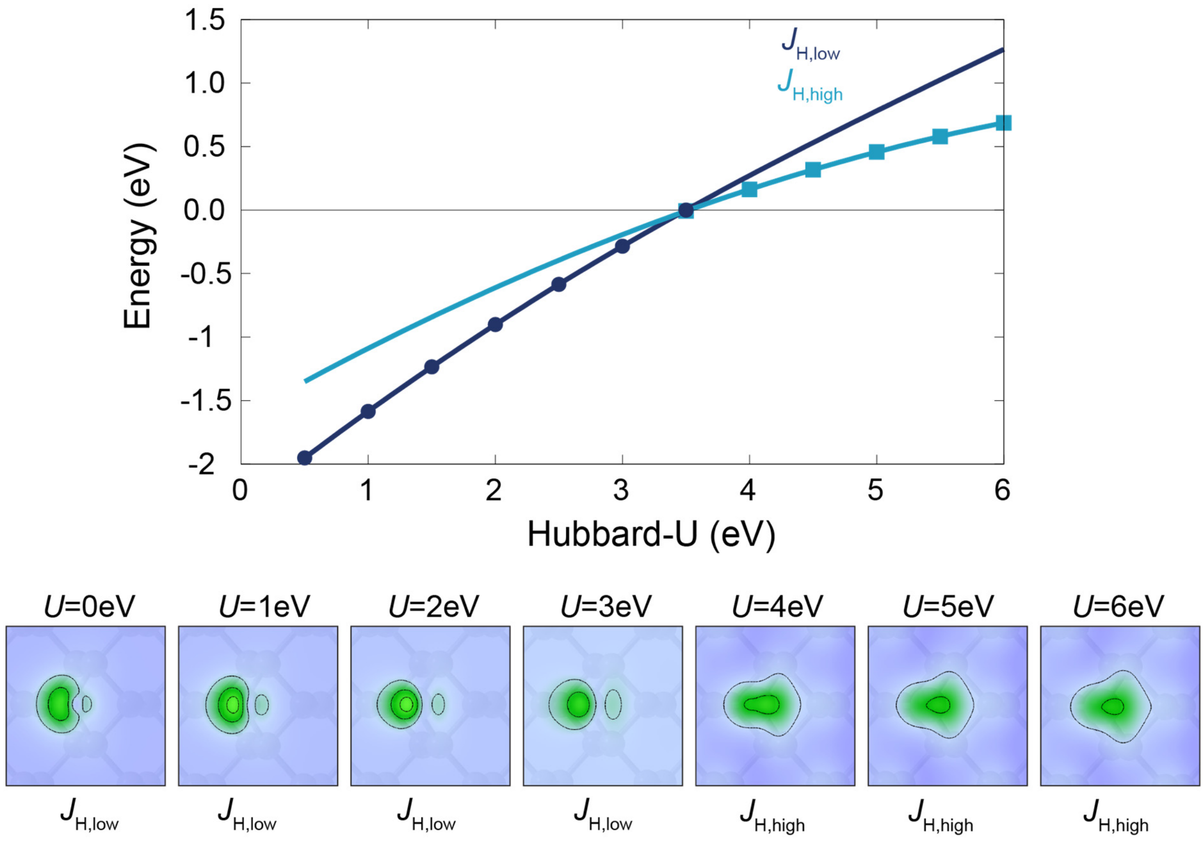


**Supplementary Figure 5. Calculations with varying Hubbard-U correction.** (upper) Plot showing total energy (markers) of $J_{H,low}$ state (dark blue) for $U$ < 3.5 eV and $J_{H,high}$ (light blue) for $U$ > 3.5 eV. Solid lines show fits to the markers. The state favorability inverts at U = 3.5 eV, where both states are stable and nearly degenerate in energy. (lower) Plots of calculated charge density distributions calculated for varying Hubbard-U parameters.

In Supplementary Figure 6, we show spin-resolved density of states (DOS) calculated at the density functional theory (DFT) level for the three states of cobalt atom on BP discussed in the paper. For the $J_T$ and $J_{H,low}$ states, sharp peaks in the vicinity of the Fermi level are visible, originating from cobalt *3d* states. Majority (spin-up) states are fully occupied in both cases, whereas minority (spin-down) states have at least one unoccupied *3d*-band, giving rise to a magnetic moment of at least 1 $\mu_B$. The contribution of Co *4s* electrons to the magnetic moment is negligible, as can be seen from the DOS projected onto *4s* states. The case of the $J_{H,high}$ state is different due to additional on-site Coulomb repulsion applied in the calculations in the form of a Hubbard-U correction. In this case, filling of minority *3d* states is energetically less favorable,

leading to a considerable energy separation between the occupied and unoccupied states. This also results in a larger imbalance between the occupied majority and minority states, increasing the magnetic moment of the *3d*-shell. One can also see that cobalt *3d* states are broadened, which may be associated with a stronger hybridization with phosphorus states. It is worth noting that the weight of cobalt states near the valence and conduction band edges for $J_{H,high}$ is substantially smaller than for the $J_T$ and $J_{H,low}$ states. A more detailed energy spectrum calculated for the different cobalt states can be seen in Supplementary Figure 7, where *k*-resolved band structure projected onto both majority and minority Co *3d* states is shown.

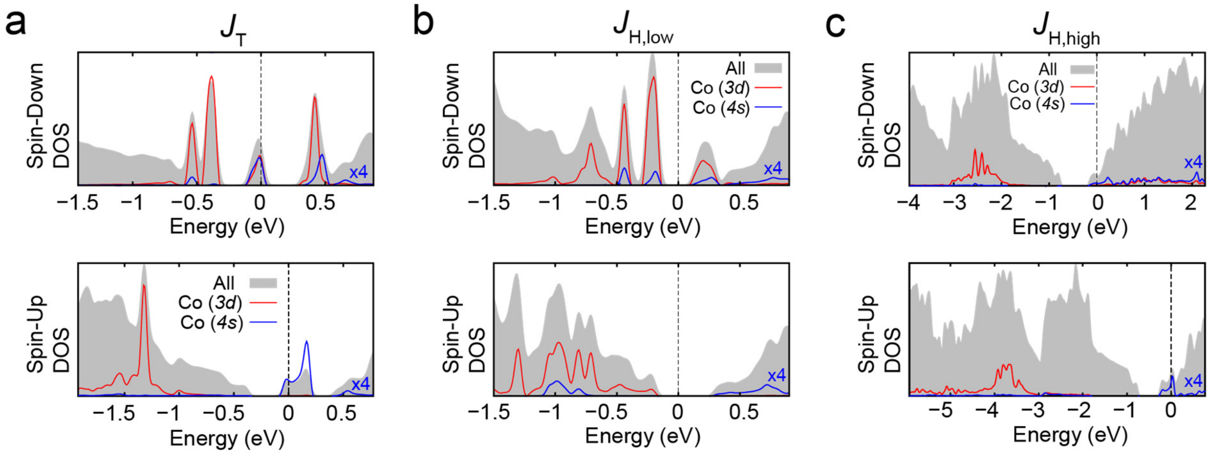


**Supplementary Figure 6. Electronic density of state calculations.** Spin-resolved DOS calculations for Co + BP with $J_T$ (left), $J_{H,low}$ (middle), and $J_{H,high}$ (right). Red curves represent Co *3d* orbital contributions, blue curves show Co *4s* orbital contributions (magnified by a factor of 4), and gray show total DOS.

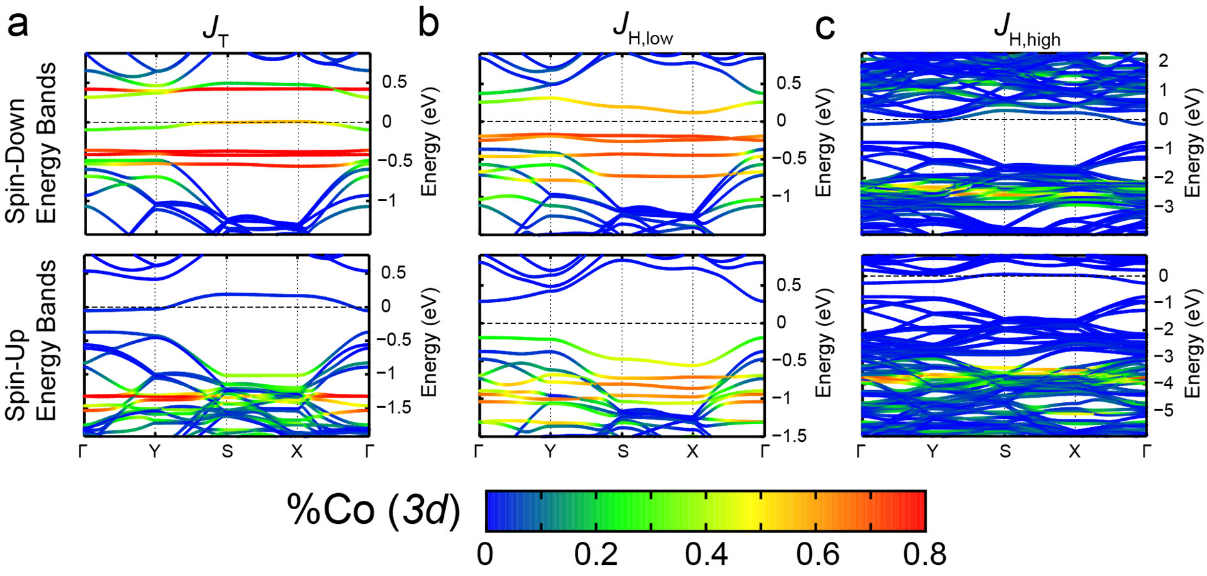


**Supplementary Figure 7. Band structure of Co on BP.** Spin-resolved calculations for Co + BP with $J_T$ (left), $J_{H,low}$ (middle), and $J_{H,high}$ (right). Color scale indicates percentage of Co *3d*-orbital contribution to each band.

In Supplementary Table 1, we list the spin-resolved *3d*-subshell occupancies of the Co adatom on BP calculated for the two orbital configurations. From the projections, it is clear a major orbital redistribution takes place within the $3d_{xz}$ and $3d_{yz}$ subshells upon vertical relaxation ($\Delta n \approx 0.4$ $e^-$). Additionally, the reduced ligand interactions make the $3d_{z2}$ subshell more favorable as the atom relaxes away from the BP surface. One can see that in the $J_{H,low}$ state the magnetic moment originates predominantly from the $3d_{x2-y2}$ orbital with some contribution from the $3d_{z2}$ orbital. On the contrary, the magnetization in the $J_{H,high}$ state is distributed almost equally over all orbitals of the *3d* symmetry except $3d_{z2}$, whose contribution is negligible.

| | $J_{H,low}$ | | | | $J_{H,high}$ | | | |
|---|---|---|---|---|---|---|---|---|
| | Spin-up *n* | Spin-down *n* | Total *n* | Magnetic Moment | Spin-up *n* | Spin-down *n* | Total *n* | Magnetic Moment |
| $3d_{z^2}$ | 0.945 | 0.785 | 1.73 | 0.16 | 0.991 | 0.962 | 1.953 | 0.029 |
| $3d_{xy}$ | 0.932 | 0.89 | 1.822 | 0.042 | 0.99 | 0.685 | 1.675 | 0.305 |
| $3d_{x^2-y^2}$ | 0.95 | 0.493 | 1.443 | 0.457 | 0.995 | 0.647 | 1.642 | 0.348 |
| $3d_{xz}$ | 0.928 | 0.883 | 1.811 | 0.045 | 0.972 | 0.404 | 1.376 | 0.568 |
| $3d_{yz}$ | 0.944 | 0.873 | 1.817 | 0.071 | 0.986 | 0.496 | 1.482 | 0.49 |
| $3d_{tot}$ | 4.699 | 3.924 | 8.623 | 0.775 | 4.934 | 3.194 | 8.128 | 1.74 |
| $4s$ | 0.124 | 0.117 | 0.241 | 0.007 | 0.266 | 0.116 | 0.382 | 0.15 |
| $4s+3d_{tot}$ | 4.823 | 4.041 | 8.864 | 0.782 | 5.2 | 3.31 | 8.51 | 1.89 |

**Supplementary Table 1. Projections onto the cubic harmonics.** Electronic charge (spin up, spin, down, and total) and magnetic moment projected onto the cubic harmonics for $J_{H,low}$ and $J_{H,high}$. Due to differences in orthogonality between the original *d*-orbital hybrid functions and the cubic harmonics, the total *d*-orbital occupancy is slightly overestimated with respect to the populations given in figure 2.

To gain insight into the charge redistribution around the Co adatom on the BP surface, we calculate the in-plane averaged charge density difference (*Δn)* along the z-direction. Here*, Δn* is defined as $\Delta n = n_{Co+BP} - n_{BP} - n_{Co}$, where $n_{Co+BP}$ is the charge density of BP with the Co adatom, $n_{BP}$ is the charge density of BP without the adatom, and $n_{Co}$ is the charge density of the free Co atom in a S = 3/2 state. The corresponding quantity (*Δn)* is shown in Supplementary Figure 8 for the two orbital configurations of the Co atom on BP. One can see that the charge in the adatom tends to be localized toward the surface plane causing perturbations of the charge density within the surface. At the same time, no significant charge transfer is observed between Co and BP. The primary difference between the two states is the spatial extension of the charge redistribution, which is greater for $J_{H,high}$. An area of comparison is highlighted in purple in Supplementary Figure 8a, where the *Δn* for the two states is directly compared. These observations are consistent with the subshell projections from Supplementary Table 1

(increased charge in the $3d_{z2}$ subshell for $J_{H,high}$), and add further evidence (in addition to the broadened linewidths of Supplementary Figure 6) for stronger hybridization in $J_{H,high}$.

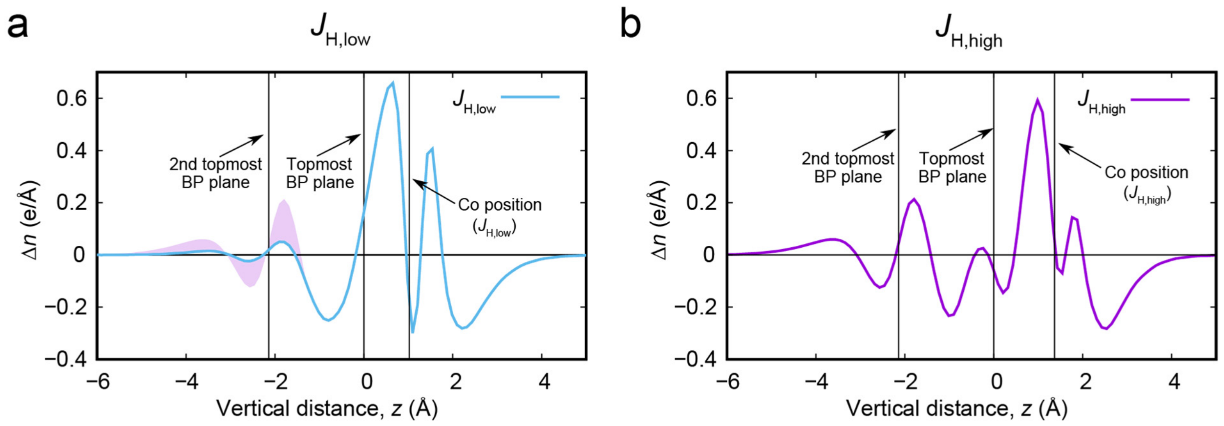


**Supplementary Figure 8. Out-of-plane charge distribution.** Charge redistribution (*Δn)* defined in the text for (a) $J_{H,low}$ and (b) $J_{H,high}$. Free Co atom positions are taken from relaxed DFT calculations for $J_{H,low}$ and $J_{H,high}$. Purple shaded area in (a) is a direct comparison of *Δn* for $J_{H,low}$ and $J_{H,high}$ near the lower plane of phosphorus atoms.

Magnetic anisotropy has been calculated for $J_{H,low}$ and $J_{H,high}$ states using DFT taking into account spin-orbit coupling, with the results given in Supplementary Table 2. The anisotropy is defined as $\Delta E = E_{easy} - E_{hard}$, where $E_{easy}$ and $E_{hard}$ are the total energy calculated when magnetic moments are aligned along the easy and hard axis, respectively. Here *y* denotes the [100] direction, and *x* the [010] direction. For both orbital states the hard axis corresponds to the zigzag crystallographic direction [010]. Conversely, the the easy axis for the two states are different, which is the armchair [100] and out-of-plane (z-direction) for $J_{H,low}$ and $J_{H,high}$, respectively. The corresponding anisotropy energies amount to 0.5 meV ($J_{H,low}$) and 0.4 meV ($J_{H,high}$). The fact that the preferential orientation of the magnetic moment is different for the two orbital states is interesting, as this implies the magnetic anistropy can be controlled electrically. The anisotropy energies are generally quite small due to the relatively low-Z phosphorus atoms of the substrate; however, such energies are accessible at temperatures below 1K. Thus spin-

averaged and spin-sensitive techniques can potentially be used to investigate the magnetic properties of both $J_{H,low}$ and $J_{H,high}$.

| | $J_{H,low}$ | $J_{H,high}$ |
|---|---|---|
| $\Delta E(z\text{-}y)$ | 0.31 meV | -0.11 meV |
| $\Delta E(x\text{-}y)$ | 0.49 meV | 0.29 meV |

**Supplementary Table 2. Anisotropy Calculations.** Including spin-orbit coupling into the spin-resolved DFT calculations reveals that the $J_{H,low}$ state has easy plane anisotropy, while the $J_{H,high}$ state has easy axis anisotropy.

## Supplementary Note 3: Comparison of d*I*/d*V* spectroscopy with density of states calculations

Representative d*I*/d*V* spectra for the $J_T$ site (Fig. 2j), $J_{H,low}$ (Fig. 2k), and $J_{H,high}$ (Fig. 2l) all show strong deviation from the bulk BP[5]. Comparing these spectra to calculations for Co on single layer BP, sharp peaks in the d*I*/d*V* spectra can be attributed to Co-related impurity states or ionization events, similar to previous observations in bulk semiconductors[6]. The two peaks observed in the $J_T$ spectrum (Fig. 2j) can be assigned to ionization events, as they shift strongly (>100 mV) with varying tip height (Supplementary Figure 9a,d). The primary features in the $J_{H,low}$ spectrum, peaks at -580 mV and 280 mV (Fig. 2k), do not shift significantly with tip height (Supplementary Figure 9b,e). The predicted DOS for $J_{H,low}$ shows Co hybrid *d*-bands at both of these energies, which can tentatively be assigned to the origin of the d*I*/d*V* peaks. The measured d*I*/d*V* spectrum for $J_{H,high}$ is given in Fig. 2l. The spectrum is characterized by its diminished intensity and single sharp peak at approximately 420 mV. As seen in Supplementary Figure 10g, this peak is related to the ionization of the $J_{H,high}$ state at this bias. The relatively weak total signal and in-gap conduction (Supplementary Figure 9c, f) are in good agreement with DFT calculations (Supplementary Figure 6c) showing a shift (and reduction) of the band gap with minimal Co DOS in the vicinity of $E_F$.

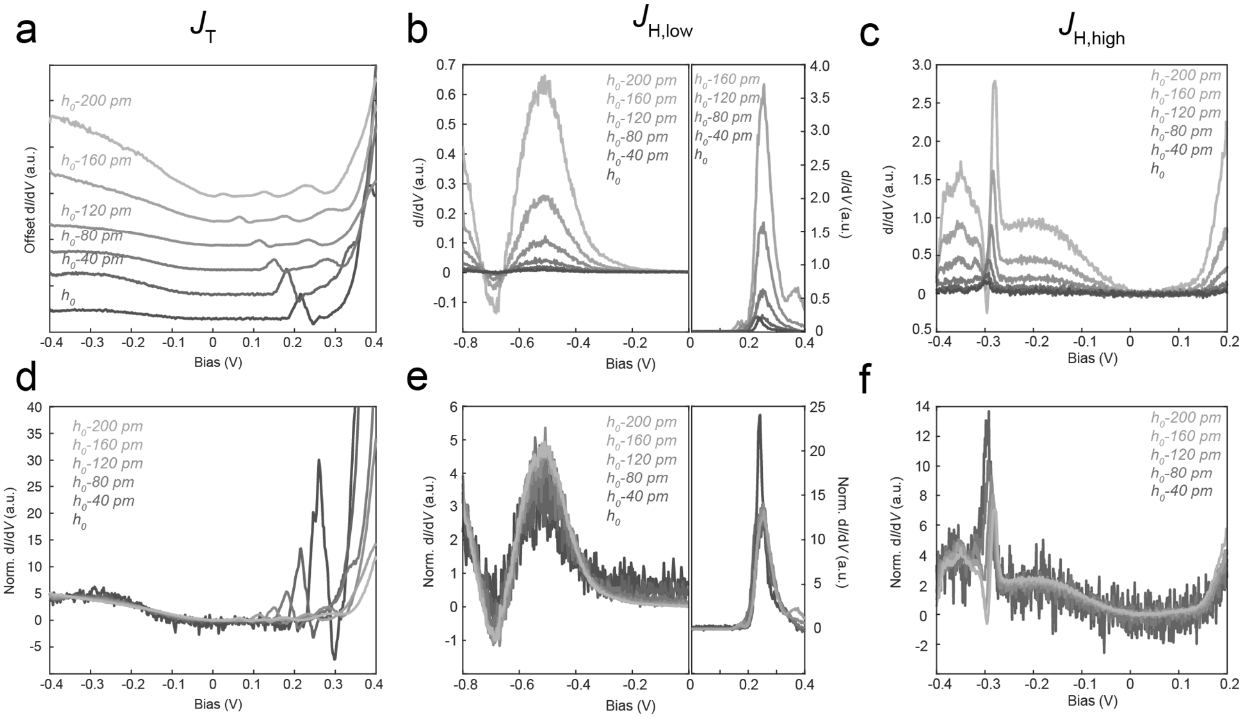

**Supplementary Figure 9. Setpoint-dependent point spectroscopy.** (a) d$I$/d$V$ spectroscopy for the $J_T$ Co atom at various heights indicated in the plot. The nominal height ($h_0$) was set at feedback parameters of $V_s$ = -400 mV, $I_t$ = 20 pA; negative distances are closer to the sample. (b) Two sets of height-resolved d$I$/d$V$ spectroscopy of the $J_{H,low}$ state: one in the valence band, the other in the conduction band. (c) d$I$/d$V$ spectroscopy of the $J_{H,high}$ state at various heights. (d-f) Spectra from (a-c) normalized by a constant proportional to the integrated d$I$/d$V$ signal over the displayed spectral window.

## Supplementary Note 4: Tip-induced ionization

Tip-induced band-bending (TIBB) locally influences the energy of semiconductor bands due to insufficient screening from charge carriers; it has been observed that if a defect level, shifted with the material bands, passes through $E_F$, it will undergo an ionization event. The instantaneous change in charge state is observable as a step in the tunneling current (peak in d$I$/d$V$) because the local potential landscape is modified via an effective Coulomb potential at the atom[6-9]. Using d$I$/d$V$ spectral mapping, we map out the spatial location of such a charging event for $J_{H,low}$ and $J_{H,high}$ in Supplementary Figure 10a-c, measuring at constant height and the specified bias voltage. The corresponding current images are shown in Supplementary Figure

10d-f. The sizes of the isotropic ionization-related events, or charging rings, scale with applied bias (Supplementary Figure 10g) roughly following hyperbolic contours of constant TIBB.

As seen in Supplementary Figure 10g, $J_{\mathrm{H,low}}$ and $J_{\mathrm{H,high}}$ show charging rings from 200 mV < $V_s$ < 700 mV, which follow similar scaling as a function of applied bias. From this, we are able to deduce that the local 3*d*-state of the Co atom does not strongly influence the electric-field screening of the tip potential. Supplementary Figure 10g also shows the bias dependence of a charging event between -300 mV < $V_s$ < -100 mV observed only for $J_{\mathrm{H,high}}$. The event clearly falls on a distinct TIBB contour and as the contour retains positive curvature, the bias range probed (-250 mV to 0 mV) must be above the flat band condition (Fig. 3d) dictated by the tip and sample work functions. Such a condition ($V_{FB} \lesssim$ -300 mV) is achievable with a tip work function of 4.0-4.1 eV. Similar flat band conditions were observed with multiple tips after preparation and calibration on Au(111). The interpretation of $V_{FB} \lesssim$ -300 mV, is further corroborated by the inverted current response of the $V_{\mathrm{s}}$ < 0 V ionization event (Supplementary Figure 10f). As the band bending is still upward in this bias regime, the ionization event is the result of a local state being moved above $E_F$; however, as $V_{\mathrm{s}}$ < 0 V electron current is passing from filled sample states to empty tip states. The loss of one additional filled state (upon ionization) reduces the number of available states contributing to the tunneling current, thereby causing a step-wise decrease in $I_{\mathrm{t}}$ upon ionization. Thus, the ionized region for $J_{\mathrm{H,high}}$ at $V_{\mathrm{s}}$ < 0 V appears as a disk-like depression (highlighted by the arrow in Supplementary Figure 10f) in the current map, whereas it appears as a disk-like protrusion for both $J_{\mathrm{H,low}}$ and $J_{\mathrm{H,high}}$ at $V_{\mathrm{s}}$ > 0 V (Supplementary Figure 10d,e). Knowledge of the flat band condition necessitates the location of the responsible states below $E_F$, as they must be ionized through upward band bending. This information, in addition to the relative radii for each charging ring, inform the qualitative energy level structure shown in Supplementary Figure 10h. From this data, it is clear that Co states near the VB edge

(Supplementary Figure 6) are ionized through upward TIBB; further quantification of the relevant energies is possible via TIBB simulations[10] extended to account for the BP band structure.

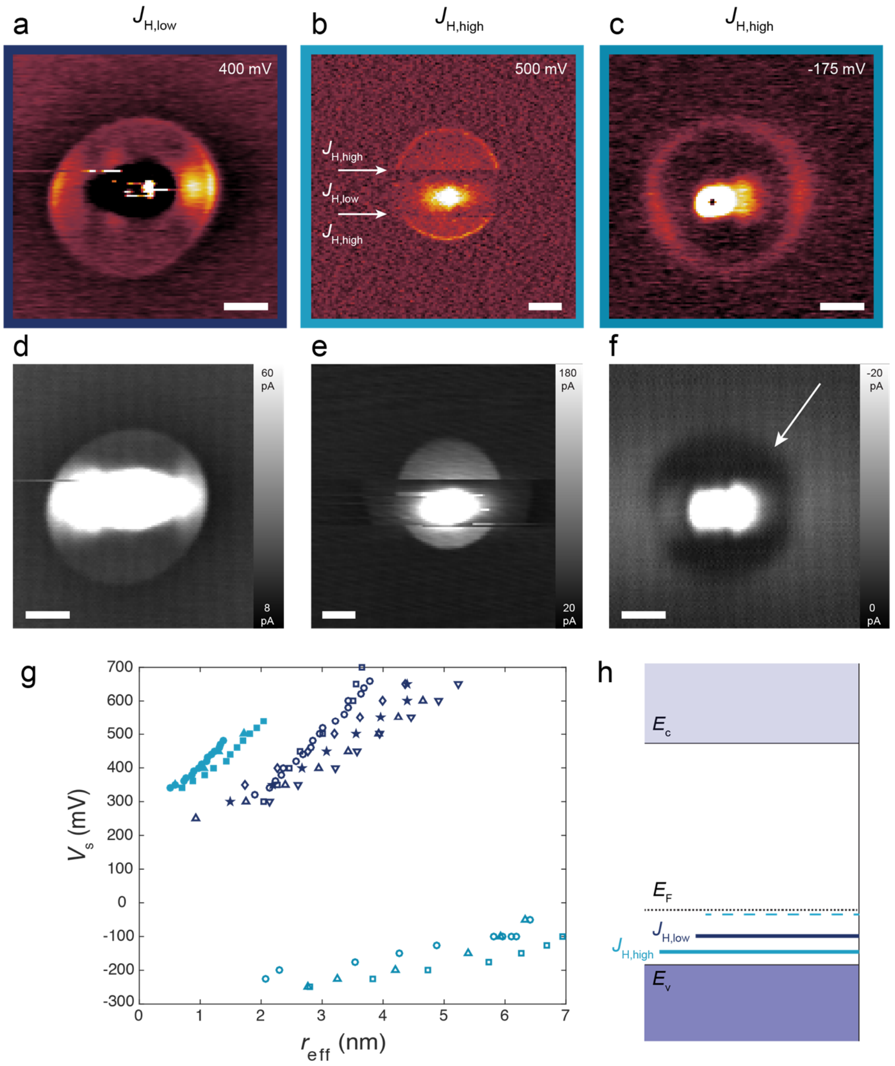


**Supplementary Figure 10. Charging ring maps.** Simultaneously acquired constant-height (a) d*I*/d*V* and (d) current maps of a $J_{H,low}$ Co atom at $V_s$ = 400 mV. Constant-height (b) d*I*/d*V* and (e) current maps of the $J_{H,high}$ state at $V_s$ = 500 mV. Constant-height (c) d*I*/d*V* and (f) current maps of the $J_{H,high}$ state at $V_s$ = -175 mV. All images: scale bar = 1 nm. The decrease in current magnitude in (f), highlighted by the white arrow, is qualitatively understood as the result of a de-charging event at negative sample bias (Fig. 4e); at $V_s$ < 0 V, electrons tunnel from filled sample states into empty tip states, when a filled electronic state is depopulated, the tunneling current decreases. Such an explanation successfully accounts for the isotropic character of the charging event and the spatial evolution of the charging state with decreasing bias magnitude. (g) Evolution of charging ring radii ($r_{eff}$) with bias voltage for $J_{H,low}$ (dark blue) and $J_{H,high}$ (light blue). (h) Qualitative level structure in the flat band condition showing primary $J_{H,low}$ (solid dark blue line) and $J_{H,high}$ (solid light blue line) energy levels responsible for ionization in the regime 200 mV < $V_s$ < 700 mV. Significantly, these are the two states that are ionized during the telegraph switching. An additional $J_{H,high}$ state (dashed light blue line) is depicted, which is responsible for the charging event at -300 mV < $V_s$ < -100 mV.

Supplementary Figure 11 demonstrates the methods used to calculate the uncertainty when estimating the charging ring radii shown in Fig. 3c. First, the ring radius was approximated via $r_{eff}=L/2\pi$, where $L$ is defined at the circumference of the ring. This effective definition was used to accommodate deviations from ideal circular behavior. The error bars were then determined by one of two methods, shown in Supplementary Figure 11. The first method fit the largest and smallest possible ellipsoids to the data, calculating $r_{min}$ and $r_{max}$ as with $r_{eff}$. The second method directly measured the smallest and largest radius for each ring. The larger of the two error values was then displayed using error bars in Fig. 3c.

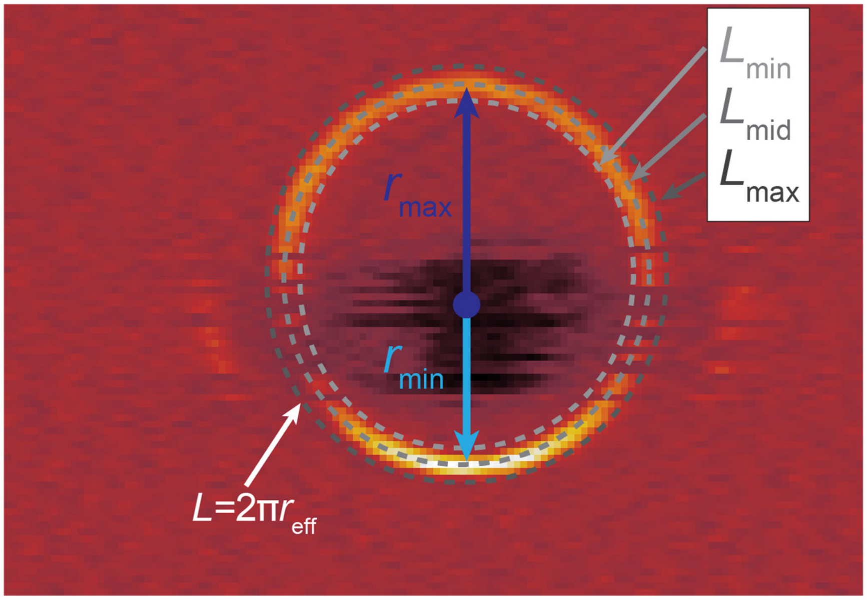


**Supplementary Figure 11. Charging ring uncertainty.** Image of charging ring observed in d$I$/d$V$ signal with two sources of experimental uncertainty shown. One source of uncertainty comes from determining the ring circumference. To estimate the possible error, the minimum and maximum possible circumferences were determined, labeled $L_{min}$ and $L_{max}$ in the figure, and used to correspondingly calculate $r_{min}$ and $r_{max}$ for a given charging ring. The second source of experimental uncertainty was introduced due to a non-spherical tip shape corresponding to a non-circular charging ring. Here, the $r_{min}$ and $r_{max}$ values were directly measured. The larger of the values from the two methods was used for error estimates.

## Supplementary Note 5: Room temperature annealing

First, a sample was dosed with Co and measured at 4.4 K (Supplementary Figure 12a – areal density of Co atoms 0.097 $nm^{-2}$). Annealing was then accomplished by removing the sample from the low-temperature STM and inserting it into a UHV sample carousel kept at room temperature for precisely 1 minute. Afterward, the sample was returned into the STM. Subsequent characterization (Supplementary Figure 12b – areal density of Co atoms 0.087 $nm^{-2}$) was conducted at 4.4 K. With the exception of some (>40%) hydrogenation (highlighted with a red circle in Supplementary Figure 12b), a large portion of the atomic species remain in either the $J_T$, $J_{H,low}$, or $J_{H,high}$ states after the anneal. A second, subsequent anneal was performed by again placing the sample into the room temperature carousel, however, the duration was increased to 10 minutes. After the second annealing, the areal density of surface species was reduced to 0.035 $nm^{-2}$ (Supplementary Figure 12c). This reduction in atomic density in conjunction with an increase in the apparent height of many species leads to the conclusion that a sizable portion of the Co atoms formed clusters. However, a number of Co species remained isolated after the annealing procedure (Supplementary Figure 12b); two $J_T$ atoms are highlighted with orange boxes (in total, at least six can be found in the image) and two $J_{H,high}$ atoms are indicated with dark red boxes (in total, at least 13 are found in the image). Expanded images showing each of these selected Co species are given at the bottom of Supplementary Figure 12c. The formation of Co clusters in Supplementary Figure 12c is partially attributed to the high Co density before the annealing step.

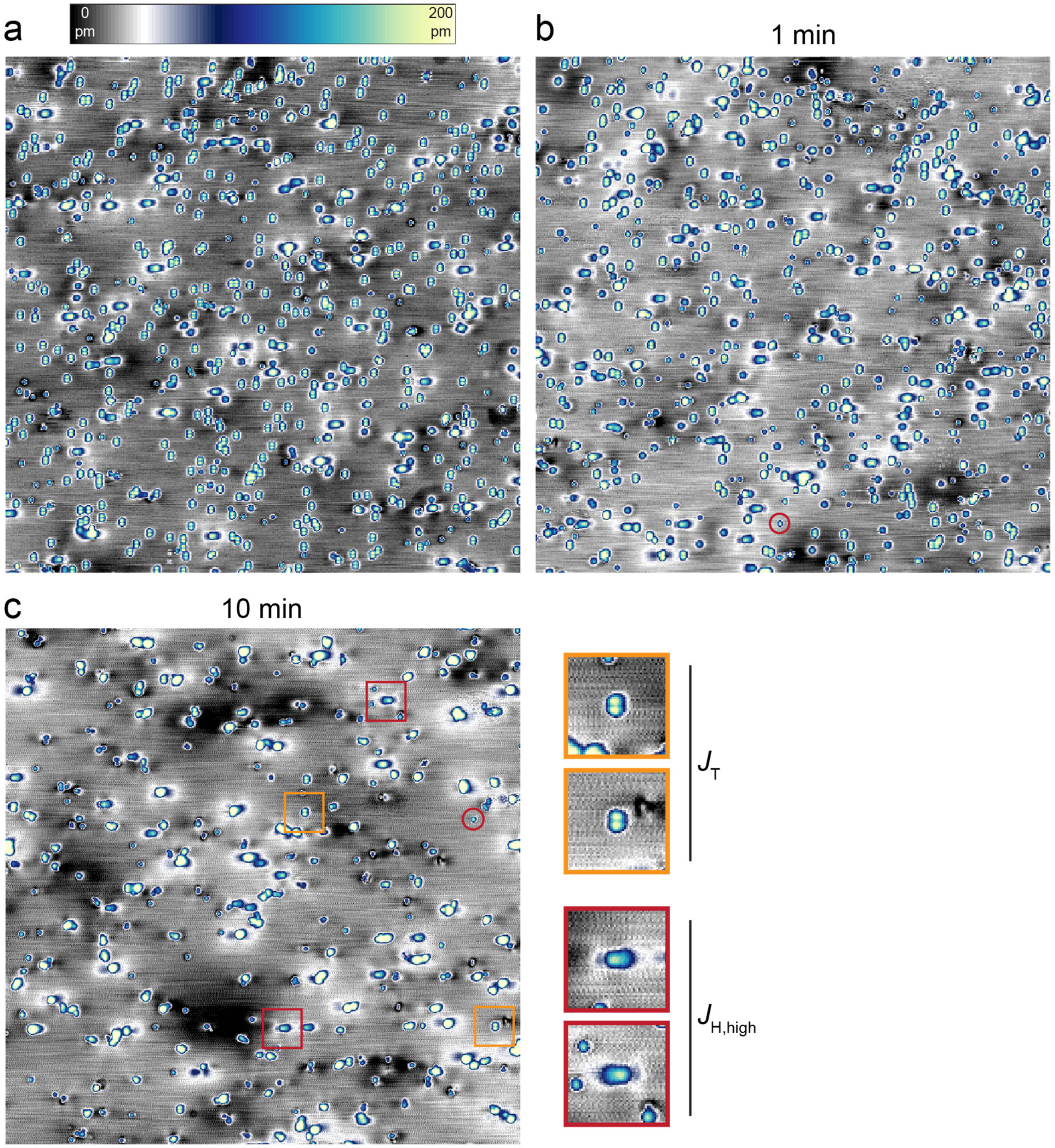


**Supplementary Figure 12. Room temperature annealing.** (a) Large area image of higher coverage (0.097 nm$^{-2}$) Co species acquired at 4.4 K before annealing ($V_s$ = -400 mV, $I_t$ = 20 pA, image = 80 nm x 80 nm). (b) Sample after annealing for 1 minute and cooling back to 4.4 K ($V_s$ = -400 mV, $I_t$ = 20 pA, image = 80 nm x 80 nm). (c) Sample after annealing for 10 minutes and cooling back to 4.4 K ($V_s$ = -400 mV, $I_t$ = 20 pA, image = 80 nm x 80 nm). Enlarged images at the bottom of (c) show isolated $J_T$ (orange boxes) and $J_{H,high}$ (red boxes) species that remain after room temperature annealing.

## Supplementary References


1 Khajetoorians, A. A. *et al.* Tuning emergent magnetism in a Hund's impurity. *Nature Nanotechnology* **10**, 958, doi:10.1038/nnano.2015.193 (2015).

2 Dubout, Q. *et al.* Controlling the Spin of Co Atoms on Pt(111) by Hydrogen Adsorption. *Physical Review Letters* **114**, 106807, doi:10.1103/PhysRevLett.114.106807 (2015).

3 Donati, F. *et al.* Magnetic Moment and Anisotropy of Individual Co Atoms on Graphene. *Physical Review Letters* **111**, 236801, doi:10.1103/PhysRevLett.111.236801 (2013).

4 Natterer, F. D., Patthey, F. & Brune, H. Quantifying residual hydrogen adsorption in low-temperature STMs. *Surface Science* **615**, 80-87, doi:https://doi.org/10.1016/j.susc.2013.04.008 (2013).

5 Kiraly, B., Hauptmann, N., Rudenko, A. N., Katsnelson, M. I. & Khajetoorians, A. A. Probing Single Vacancies in Black Phosphorus at the Atomic Level. *Nano Letters* **17**, 3607-3612, doi:10.1021/acs.nanolett.7b00766 (2017).

6 Marczinowski, F., Wiebe, J., Meier, F., Hashimoto, K. & Wiesendanger, R. Effect of charge manipulation on scanning tunneling spectra of single Mn acceptors in InAs. *Physical Review B* **77**, 115318, doi:10.1103/PhysRevB.77.115318 (2008).

7 Teichmann, K. *et al.* Controlled Charge Switching on a Single Donor with a Scanning Tunneling Microscope. *Physical Review Letters* **101**, 076103 (2008).

8 Sessi, P., Bathon, T., Kokh, K. A., Tereshchenko, O. E. & Bode, M. Single Electron Gating of Topological Insulators. *Advanced Materials* **28**, 10073-10078, doi:10.1002/adma.201602413 (2016).

9 Zheng, H., Kröger, J. & Berndt, R. Spectroscopy of Single Donors at ZnO(0001) Surfaces. *Physical Review Letters* **108**, 076801-076805, doi:10.1103/PhysRevLett.108.076801 (2012).

10 Feenstra, R. M. Electrostatic potential for a hyperbolic probe tip near a semiconductor. *Journal of Vacuum Science & Technology B: Microelectronics and Nanometer Structures Processing, Measurement, and Phenomena* **21**, 2080-2088, doi:10.1116/1.1606466 (2003).
